# Environmental Signals and Regulatory Pathways That Influence Exopolysaccharide Production in Rhizobia

**DOI:** 10.3390/ijms12117898

**Published:** 2011-11-15

**Authors:** Monika Janczarek

**Affiliations:** Department of Genetics and Microbiology, University of M. Curie-Skłodowska, Akademicka 19 st., Lublin 20-033, Poland; E-Mail: mon.jan@poczta.umcs.lublin.pl; Tel.: +48-81-537-5974; Fax: +48-81-537-5959

**Keywords:** exopolysaccharide synthesis, *exo* and *pss* genes, *Sinorhizobium meliloti*, *Rhizobium leguminosarum*, quorum sensing, motility, rhizobium-legume symbiosis

## Abstract

Rhizobia are Gram-negative bacteria that can exist either as free-living bacteria or as nitrogen-fixing symbionts inside root nodules of leguminous plants. The composition of the rhizobial outer surface, containing a variety of polysaccharides, plays a significant role in the adaptation of these bacteria in both habitats. Among rhizobial polymers, exopolysaccharide (EPS) is indispensable for the invasion of a great majority of host plants which form indeterminate-type nodules. Various functions are ascribed to this heteropolymer, including protection against environmental stress and host defense, attachment to abiotic and biotic surfaces, and in signaling. The synthesis of EPS in rhizobia is a multi-step process regulated by several proteins at both transcriptional and post-transcriptional levels. Also, some environmental factors (carbon source, nitrogen and phosphate starvation, flavonoids) and stress conditions (osmolarity, ionic strength) affect EPS production. This paper discusses the recent data concerning the function of the genes required for EPS synthesis and the regulation of this process by several environmental signals. Up till now, the synthesis of rhizobial EPS has been best studied in two species, *Sinorhizobium meliloti* and *Rhizobium leguminosarum.* The latest data indicate that EPS synthesis in rhizobia undergoes very complex hierarchical regulation, in which proteins engaged in quorum sensing and the regulation of motility genes also participate. This finding enables a better understanding of the complex processes occurring in the rhizosphere which are crucial for successful colonization and infection of host plant roots.

## 1. Introduction

Rhizobia comprise a very diverse group of nitrogen-fixing symbiotic bacteria that belong to α and β subclasses of the *Proteobacteria*, and are members of several genera, including *Rhizobium*, *Sinorhizobium*, *Mesorhizobium*, *Bradyrhizobium*, *Azorhizobium*, *Allorhizobium* and *Methylobacterium* (α*-*rhizobia), as well as *Burkholderia* and *Cupriavidus* (β*-*rhizobia) [1]. They possess the ability to associate with leguminous plants and induce the formation of special organs, termed nodules, on roots and stems, inside of which atmospheric nitrogen is reduced to ammonia by the enzymatic complex of nitrogenase. The establishment of symbiosis is a very complex process involving a coordinated exchange of signals between the host plant and its microsymbiont, in which flavonoids secreted by plant roots and rhizobial lipochitin oligosaccharides (Nod factors) play key roles [2]. In addition, both the bacterial and plant cell surface components participate in this plant-microbe interaction [3,4]. The outer surface of rhizobia consists of complex polysaccharides, among these lipopolysaccharides (LPS), capsular (CPS) and extracellular (EPS) polysaccharides, K-antigen polysaccharide (KPS), cyclic β-glucan (CG) and recently reported in *R. leguminosarum*, high-molecular-weight neutral polysaccharide (NP or glucomannan) and gel-forming polysaccharide (GPS) [5,6]. LPS, composed of lipid A, a core oligosaccharide and an antigen polysaccharide, are anchored in the outer membrane. CPS are tightly associated with the bacterial surface and are neutral or acidic polysaccharides, with a structure in most species very similar or even identical to EPS. In contrast to CPS, EPS are weakly associated with the bacterial surface and are released in large amounts into the environment [3,5]. Rhizobial K-antigens show structural analogies to group II K-antigens of *Escherichia coli* and they, in some cases, can functionally substitute for EPS [7]. Cyclic β-glucans are important for hypoosmotic adaptation of bacteria and for plant infection (in both indeterminate- and determinate-nodule-forming symbioses) [8]. CG-deficient mutants demonstrate an increased production of EPS [9]. All these surface polysaccharides play an essential role in the establishment of an effective symbiosis, especially with host plants that form indeterminate-type nodules, such as *Trifolium*, *Pisum*, *Vicia* and *Medicago* spp. [3].

A successful early interaction between symbiotic partners leads to the invasion of host plant roots, where bacteria divide within the tubular structures called infection threads and penetrate root tissues. Inside nodules, rhizobia are released from infection threads to the cytoplasm of host cells and differentiate into bacteroids, which provide the plant with a reduced form of nitrogen [2].

The ability to produce EPS is a widespread feature among bacteria. This heteropolymer performs several functions, such as nutrient gathering, protection against environmental stress and antimicrobial compounds, and attachment to surfaces [4]. In nitrogen-fixing bacteria which establish symbioses with legumes forming indeterminate-type nodules, EPS is additionally indispensable for successful infection of host plant roots [3]. EPS-deficient mutants of *Rhizobium leguminosarum* and *Sinorhizobium (Ensifer) meliloti* are impaired in nodule cell invasion, and as a consequence, in nitrogen fixation [10–12]. However, non-producing EPS mutant of *S. meliloti* Rm41 (*exoB*) induces normal nitrogen-fixing nodules on alfalfa, because K-antigen from this strain can substitute for EPS [13,14]. *Sinorhizobium fredii* strain HH103, which is closely related to *S. meliloti*, does not strictly require EPS and KPS to nodulate *Glycyrrhiza uralensis* plants forming indeterminate-type nodules [15]. On the other hand, alterations in rhizobial EPS can lead in some cases to impairment in symbiosis, in that makrosymbiont forms determinate-type nodules (*Bradyrhizobium japonicum* USDA110—soybean) [16]. These data indicate that, although EPS is an essential for a great majority of indeterminate-nodule-forming symbioses, there are some interesting examples showing differences with *R. leguminosarum* and *S. meliloti* species.

Rhizobial EPS is also very important for proper biofilm formation both on abiotic surfaces and on host plant roots, being the major component of the biofilm matrix [4,17]. Biosynthesis of EPS in rhizobia is a complex process regulated at both transcriptional and post-transcriptional levels and influenced by several nutrient and environmental conditions.

This review focuses on the genetic control of the biosynthesis of rhizobial EPS, the regulation of this process by different environmental factors and its relationship with other bacterial pathways.

## 2. Chemical Structure of Rhizobial Exopolysaccharides

Rhizobial EPS are species-specific (or even strain-specific) linear or branched heteropolymers and homopolymers which consist of repeating units containing mainly common monosaccharides, such as d-glucose, d-galactose, d-mannose, l-rhamnose, d-glucuronic acid and d-galacturonic acid, substituted with non-carbohydrate residues (e.g., acetyl, pyruvyl, succinyl and 3-hydroxybutanoyl groups) which are responsible for the acidic character of EPS [3]. Among these EPS, a high diversity in chemical structure has been described, which concerns their sugar composition and the size of their repeating units, the type of glycosidic linkages, non-carbohydrate modifications, and the degree of polymerization [5,6]. EPS are produced in two forms of different molecular masses, a high-molecular-weight (HMW) fraction containing polymers of 10^6^ to 10^7^ Da and a low-molecular-weight (LMW) fraction consisting of monomers, dimers and trimers of the repeating unit [18–20]. The LMW fraction is an active biological form of EPS indispensable for successful infection of leguminous plants forming indeterminate-type nodules.

**Figure 1:**
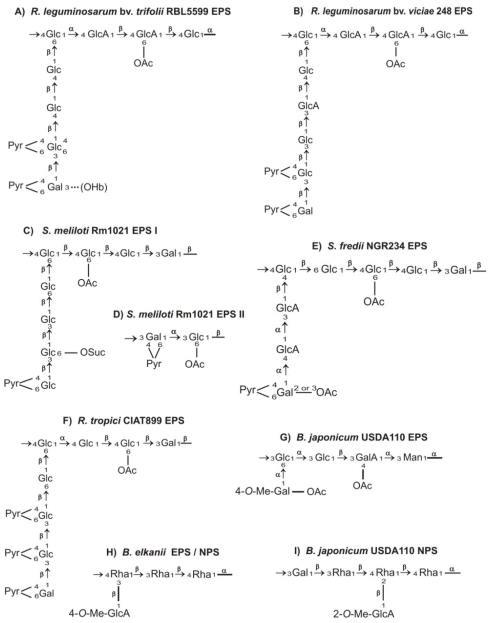
Chemical structures of the repeating units of rhizobial exopolysaccharides (EPS): (**A**) *R. leguminosarum* bv. *trifolii* [21–23], (**B**) *R. leguminosarum* bv. *viciae* [26], (**C**) *S. meliloti* EPS I [28–30], (**D**) *S. meliloti* EPS II [27,29,31], (**E**) *S. fredii* NGR234 [32], (**F**) *R. tropici* [33], (**G**) *B. japonicum* [34,35], (**H**) *B. elkanii* [36], (**I**) *B. japonicum* NPS [36–38].

In general, EPS synthesized by fast-growing rhizobia (e.g., *S. meliloti* and *R. leguminosarum*) are composed of octasaccharide repeating units, in which glucose is a dominant sugar component (Figure 1). *R. leguminosarum* strains belonging to different biovars (*trifolii*, *viciae* and *phaseoli*) and establishing symbiosis with different plant hosts (e.g., *Trifolium*, *Pisum*, *Vicia* and *Phaseolus* spp.), produce EPS which have a similar basic structure but may vary in their patterns of non-carbohydrate modification. *R. leguminosarum* EPS consists of repeating units that contain d-glucose, d-glucuronic acid and d-galactose in a molar ratio 5:2:1, joined by β-1,3 and β-1,4 glycosidic bonds, and modified by acetyl, pyruvyl and 3-hydroxybutanoyl groups (Figure 1A) [21–25]. Hovewer, *R. leguminosarum* bv. *viciae* strain 248 produces EPS whose repeating units have an extra glucuronic acid residue and absence of 3-hydroxybutanoyl group (Figure 1B) [26].

*S. meliloti*, which induces nodule formation on alfalfa (*Medicago sativa*) plants produces two structurally distinct EPS, succinoglycan (EPS I) and synthesized under phosphate starvation galactoglucan (EPS II) [27–29]. EPS I is composed of repeating units containing seven residues of d-glucose and one residue of d-galactose joined by β-1,3, β-1,4 and β-1,6 glycosidic linkages, and substituted with acetyl, pyruvyl and succinyl groups (Figure 1C) [28–30]. EPS II consists of disaccharide repeating units containing d-glucose and d-galactose in a molar ratio 1:1 linked by α-1,3 and β-1,3 bonds. Most of the glucosyl residues are 6-*O*-acetylated and all the galactosyl residues are substituted with 4,6-*O*-pyruvyl groups (Figure 1D) [29,31].

*Sinorhizobium fredii* NGR234, which has a very wide host range (it nodulates over 112 host plants), produces EPS whose repeating units contain d-galactose, d-glucuronic acid and d-glucose in a molar ratio 2:2:4, with acetyl and pyruvyl modifications (Figure 1E) [32].

EPS synthesized by *Rhizobium tropici* is composed of octasaccharide units containing d-glucose and d-galactose in a molar ratio 6:2, and modified by pyruvyl and acetyl residues (Figure 1F) [33].

In contrast to EPS of fast-growing rhizobia, the structures of EPS synthesized by slow-growing bradyrhizobia demonstrate an even higher diversity. For example, *Bradyrhizobium japonicum* EPS is composed of pentasaccharide subunits containing d-mannose, d-galacturonic acid, d-glucose and d-galactose in a molar ratio 1:1:2:1 (Figure 1G) [34,35], whereas the tetrasaccharide subunits of *Bradyrhizobium elkani* EPS contain only two sugars, L-rhamnose and 4-*O*-methyl-d-glucuronic acid (molar ratio 3:1) (Figure 1H) [36]. *B. japonicum* also synthesizes a nodule-specific polysaccharide, termed NPS, which differs from the EPS produced by these bacteria in the free-living stage (Figure 1I) [36–38]. In contrast to *B. japonicum* NPS, NPS synthesized by *B. elkani* has a structure identical to its EPS.

*Azorhizobium caulinodans*, the microsymbiont of the tropical legume *Sesbania rostrata*, produces EPS of the same structure both in culture and inside nodules. It is a linear homopolysaccharide of α-1,3-linked 4,6-*O*-pyruvyl-d-galactosyl residues [39].

## 3. Genetic Control of EPS Synthesis in Rhizobia

**Figure 2:**
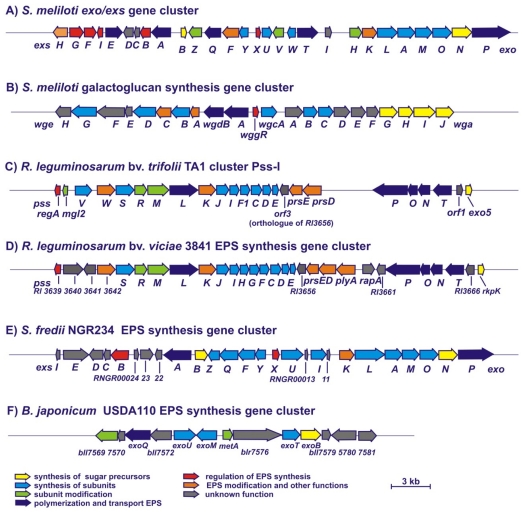
Genetic organization of EPS synthesis gene clusters in *S. meliloti* ((**A**) *exo*/*exs* cluster and (**B**) *exp* cluster involved in the synthesis of EPS I and EPS II, respectively) (Acc. no. NC_003078) [40]; (**C**) *R. leguminosarum* bv. *trifolii* TA1 (Acc. no. DQ384110) [42]; (**D**) *R. leguminosarum* bv. *viciae* 3841 (Acc. no. AM236080) [41]; (**E**) *S. fredii* NGR234 (Acc. no. AY316746); (**F**) *B. japonicum* USDA110 (Acc. no. NC_004463) [47].

Similarly to other bacteria, the synthesis of EPS in rhizobia is a multi-step process requiring the coordinated activity of many enzymatic proteins. Genes engaged in this process are usually grouped in large clusters and located on rhizobial chromosomes or megaplasmids (Figure 2) [40–47]. Among these are genes encoding enzymes indispensable for the synthesis of nucleotide sugar precursors, enzymes engaged in unit assembly and modification, and proteins responsible for polymerization of repeating units and transport of EPS outside bacteria [3].

EPS synthesis is carried out by a multi-protein complex located in both inner (IM) and outer (OM) membranes. Undecaprenol phosphate, anchored in the inner leaflet of the IM, is a sugar acceptor for the synthesis of EPS and other bacterial heteropolysaccharides. Nucleotide diphospho-sugars serving as precursors are sequentially bonded to the acceptor by specific glycosyl transferases, resulting in growing polysaccharide subunits. Then, the subunits are flipped across the IM to the periplasmic space by the action of a Wzx-like translocase [48,49]. Polymerization of repeating units is most probably coupled with export of the growing chain of the polymer to the bacterial surface. This process involves the activity of a Wzy-like polymerase and a Wzc-like inner membrane-periplasmic auxiliary protein [48,50].

### 3.1. Genes Involved in the Synthesis of *S. meliloti* EPS I and EPS II

Up to now, the biosynthesis of rhizobial EPS and regulation of this process has been best studied for succinoglycan (EPS I) of *S. meliloti*. The data concerning the synthesis of EPS in other rhizobial species are much more fragmentary. In *S. meliloti*, the genes involved in the synthesis of EPS I form a large *exo*/*exs* cluster (~35 kb) located on the pSymB megaplasmid (Figure 2A) [30,40,51–56]. In this region, 28 *exo*/*exs* genes organized in several operons have been identified, among them the genes encoding enzymes for the synthesis of nucleotide sugar precursors (*exoB* and *exoN*), enzymes involved in unit assembly (*exoY*, *exoF*, *exoA*, *exoL*, *exoM*, *exoO*, *exoU* and *exoW*) and modification (*exoZ*, *exoH* and *exoV*), and proteins responsible for polymerization of repeating units and transport of EPS I (*exoP*, *exoT*, *exoQ* and *exsA*) [51–59]. Moreover, other genes essential for sugar precursor synthesis (*exoC*) and regulation of EPS I production (*exoD*, *exoR*, *exoS* and *mucR*) are not linked with this region, but dispersed throughout the chromosome of *S. meliloti* [60–64]. Proteins encoded by the *exoC*, *exoN* and *exoB* genes are engaged in the synthesis of nucleotide sugar precursors (UDP-glucose and UDP-galactose). ExoC phosphoglucomutase converts glucose-6-phosphate into glucose-1-phosphate [64]. ExoN possesses a UDP-glucose pyrophosphorylase activity and synthesizes UDP-glucose from glucose-1-phosphate [52,54]. ExoB is a UDP-glucose 4-epimerase which synthesizes UDP-galactose from UDP-glucose [65]. A mutation in *exoN* only results in the production of diminished amounts of EPS I, whereas mutations in both *exoC* and *exoB* genes abolish the synthesis of this polymer and affect the production of other polysaccharides, such as EPS II and LPS, which contain galactose [52,54].

The assembly of octasaccharide repeating units of EPS I is initiated by the addition of UDP-galactose to the lipid carrier located in the IM. This step engages two proteins, galactosyl-IP-transferase encoded by *exoY* and auxiliary ExoF protein [56,62]. The next steps of subunit synthesis are carried out by six glucosyltransferases encoded by *exoALMOUW* genes which add sequential residues of glucose to the growing chain of the subunit [30,52,54,55]. However, the gene encoding the enzyme responsible for the addition of the last glucose to the subunit has not yet been identified. A mutation in the *exoY* gene resulted in a very strong symbiotic effect; a strain carrying this mutation was totally defective in the production of EPS I and formation of infection threads and, consequently, in nitrogen fixation [52,56]. Also, mutants in the *exoA*, *exoL* and *exoM* genes produced no detectable amounts of EPS I and formed nodules inefficient in nitrogen-fixation [11,54].

The protein products of three *exo* genes, *exoZ*, *exoH* and *exoV*, are needed for the addition of non-sugar modifications to the subunits; ExoZ is involved in the addition of acetyl, ExoH provides succinyl, and ExoV pyruvyl groups (Figure 2A) [11,30,55,65]. An *exoV* mutant accumulates only monomer units, indicating that the presence of a pyruvyl substituent in the units is crucial for the polymerization and secretion of EPS I. On the other hand, succinyl groups seem to be significant for the formation of the LMW fraction of this polymer. The function of this type of modification is confirmed by the phenotype of the *exoH* mutant, which exclusively synthesizes HMW EPS I lacking succinyl groups and induces the formation of ineffective nodules without bacteria on alfalfa. The absence of acetyl groups in EPS I causes the weakest symbiotic effect among all types of non-sugar substituents, as an *exoZ* mutant forms nitrogen-fixing nodules on its host plant and exhibits only a slightly diminished efficiency in the formation of infection threads [11,30].

Polymerization and export of EPS I are carried out by proteins encoded by *exoPQT* genes (Figure 2A) [51,54,59]. In particular, ExoP, an autophosphorylated tyrosine kinase has a crucial role in this process. A mutation of *exoP* blocks polymerization of repeating units. Recently, Jofre and Becker [59] have established that the *N*-terminal domain of ExoP, located mainly in the periplasmic space, is essential for EPS I synthesis. A deletion of the *C*-terminal domain of this protein, displaying an ATPase activity, resulted only in a decreased production of EPS I, confirming rather a regulatory function of this domain [20,51,58]. Two other proteins engaged in polymerization are ExoQ, responsible for the production of HMW EPS I, and ExoT, participating in the biosynthesis of its LMW form (trimers and tetramers of octasaccharide units).

Furthermore, ExsA protein belonging to ABC transporters is also important for the secretion of HMW EPS I [57]. An LMW form of this heteropolymer, which is especially active in symbiosis, is also generated as a result of a cleavage of its HMW fraction by two distinct enzymes, ExoK (β-1,3-1,4-glucanase) and ExsH (succinoglycan depolymerase). Acetyl and succinyl modifications present in EPS I influence the susceptibility of this polysaccharide to cleavage by these glycanases [66].

The synthesis of the second *S. meliloti* exopolysaccharide, named galactoglucan (EPS II), is directed by *exp* genes located in a 27-kb cluster on the pSymB plasmid, at a distance of 160 kb from the *exo*/*exs* genes (Figure 2B) [67,68]. This cluster contains 22 genes organized into five operons: *wga* (*expA*), *wgcA* (*expC*), *wggR* (*expG*), *wgd* (*expD*) and *wge* (*expE*) [69]. Among them, four genes (*wgaG*, *wgaH*, *wgaI* and *wgaJ*) are involved in the synthesis of deoxythymidine diphospho-sugar precursors (dTDP-rhamnose and dTDP-glucose), and six genes encode potential glycosyltransferases: WgaB and WgeB β-glucosyltransferases and WgaC, WgcA, WgeD and WgeG galactosyltransferases. Other genes of this cluster are potentially engaged in the polymerization (*wgdA* and *wgdB*) and regulation of EPS II synthesis (*wggR*) [67–69].

### 3.2. Genes Involved in the Synthesis of EPS in *R. leguminosarum*

In contrast to the well-studied model of the synthesis of *S. meliloti* EPS I, the data concerning EPS biosynthesis in *R. leguminosarum* are much more scarce. Genes involved in the synthesis of nucleotide sugar precursors as well as genes engaged in the synthesis and export of EPS are located on the chromosome of *R. leguminosarum*, and the majority of them are grouped in a large cluster termed Pss-I (Figure 2C) [42].

So far, two genes, *exoB* and *exo5*, responsible for the synthesis of sugar precursors have been characterized in *R. leguminosarum. exoB* encodes a UDP-glucose 4-epimerase indispensable for the synthesis of UDP-galactose, which is a donor of this sugar in the synthesis of EPS and other polysaccharides containing galactose [70,71]. An *exoB* mutant produces EPS lacking terminal galactoses in the repeating units and is almost completely unable to invade host plant roots, inducing the formation of abnormal nodules. The *exo5* gene, located in the cluster Pss-I, codes for a UDP-glucose dehydrogenase which is responsible for the conversion of UDP-glucose to UDP-glucuronic acid (Figure 2C). An *exo5* mutant displays pleiotropic effects, including changes in the bacterial cell envelope and defectiveness in symbiosis [72]. This mutant is able to synthesize neither UDP-glucuronic nor UDP-galacturonic acids and, as a consequence, does not produce EPS and CPS, and its LPS lacks galacturonic acid [72,73].

The *pssA* gene encoding a protein participating in the first step of the synthesis of the octasaccharide subunit represents a single open reading frame and is located at a long distance from other *pss* genes. This gene encodes an integral membrane-bound protein of glucosyl-IP-transferase activity, which transfers glucose-1-phosphate from UDP-glucose to the lipid carrier [74,75]. *pssA* is a very conserved gene present in all *R. leguminosarum* biovars [12,74,76,77] and also in other closely related species, such as *Rhizobium etli* and *Rhizobium gallicum* [78]. Mutations in *pssA* totally abolish EPS production and result in the induction of empty (devoid of bacteria) non-nitrogen-fixing nodules on roots of host plants which form indeterminate-type nodules (clover, pea and vetch) [10,12,79]. The transcription of *pssA ex planta* is at a very low level, suggesting that the expression of this key gene for EPS synthesis is under very stringent regulation [80]. Also inside nodules, *pssA* expression was at a very low, almost undetectable level [81–83].

The subsequent steps of the assembly of the repeating unit engage proteins encoded by *pss* genes located in the cluster Pss-I (Figure 2C). This 35-kb long region, encompassing more than 20 genes, is highly conserved among all the so far sequenced genomes of *R. leguminosarum* strains [41–45,84] and the closely related *R. etli* [43]. The addition of a second sugar residue to the subunit is catalyzed by a glucuronosyl-β-1,4-glucosyltransferase encoded by two genes of this region, *pssD* and *pssE* [12,75]. A *pssD* mutant demonstrates a very similar phenotype to the *pssA* mutant; it does not produce EPS and elicits non-nitrogen-fixing nodules on host plants, which contain almost no bacteria. Mutations in the *pssDE* and even more pronouncedly, in the *pssA* gene, exhibit also other effects, including differences in the levels of the synthesis of several proteins [77,85] and LPS profiles in comparison to the parental strain [86,87].

The third step of repeating unit synthesis is carried out by a glucuronosyl-β-1,4-glucuronosyltransferase encoded by *pssC* [12,75,84]. A mutation in this gene results in more than a 2-fold decrease of EPS production and induction of nitrogen-fixing nodules on clover [12], whereas failed nodulation on vetch [84].

So far, enzymes participating in the next steps of the synthesis of the EPS subunit have not been characterized, although it is very probable that some of the *pss* genes located in the Pss-I cluster, which encode putative glycosyltransferases (e.g., *pssF*, *pssG*, *pssH*, *pssI*, *pssJ* and *pssS*) might be engaged in this process (Figure 2C,D) [42,84]. Other *pss* genes present in this cluster, such as *pssR*, encoding a putative acetyltransfease, and *pssM*, encoding a ketal pyruvate transferase, are most probably involved in the addition of non-sugar substituents to EPS subunits. Recently, the role of the *pssM* gene in EPS modification and the symbiosis of *R. leguminosarum* with pea plants has been confirmed [88]. A mutation of this gene led to the absence of pyruvyl groups at the sub-terminal glucose in repeating units. As a result of this change in the composition of EPS, the bacteria elicited non-nitrogen-fixing nodules on the host plant. Nodules induced by the *pssM* mutant showed normal nodule invasion and release of bacteria into plant cells, but their differentiation into bacteroids was impaired [88]. These findings confirm that this type of modification of EPS in *R. leguminosarum* is crucial for its biological function in the establishment of an effective symbiosis.

Furthermore, in *R. leguminosarum* bv. *viciae*, the *exo-344* gene encoding glycosyltransferase responsible for the addition of the galactose residue was identified, which could be engaged in the last step of the assembly of the subunit [23]. An *exo-344* mutant demonstrates a phenotype very similar to the *exoB* mutant, as it produces only monomer units lacking the terminal galactose.

Polymerization and the export of EPS outside bacteria are carried out by a secretion system consisting of at least three proteins encoded by the *pssT*, *pssN* and *pssP* genes of the *pssTNOP* operon (Figure 2C) [42]. The PssP protein, displaying a significant identity to *S. meliloti* ExoP and other membrane-periplasmic auxiliary proteins involved in the synthesis of HMW EPS I and CPS, is the major component of this system [89]. Similarly to the phenotype of the *S. meliloti exoP* mutant, the deletion of *pssP* totally abolishes EPS production in *R. leguminosarum*. PssT is an integral inner membrane protein similar to Wzy-like proteins, and a mutation in this gene results in overproduction of EPS [90]. A third component of this secretion system, the PssN lipoprotein, is an outer membrane-associated protein directed to the periplasmic space by its *N*-terminal signal sequence [91]. Additionally, the PssL protein, which displays a significant similarity to Wzx-type flippases participating in the translocation of the O-antigen from the inner to the outer leaflet of the IM, might also be a component of this complex [92].

*pssO*, another gene of the *pssTNOP* operon, encodes a protein uniquely found in *R. leguminosarum* and *R. etli* species, which is secreted outside of bacteria and remains attached to cells [93]. A *pssO* mutant does not produce EPS and elicits nodules inefficient at fixing nitrogen on clover plants, indicating that this protein is important for the synthesis and/or transport of EPS, although its function in this process has not been established precisely.

Apart from the *pss* genes mentioned above, other genes essential for EPS synthesis but not directly engaged in this process are also present in the Pss-I cluster. These include the *plyA* and *prsDE* genes located close to *pssCDE* (Figure 2C,D) [42]. The *prsDE* genes encode components of the type I protein secretion system, which is conserved in all *R. leguminosarum* biovars, as well as in *R. etli*, *S. meliloti* and *Agrobacterium tumefaciens* [4]. This secretion system exhibits an atypical broad substrate specificity, exporting at least 13 substrates, among them glycanases (PlyA, PlyB and PlyC), rhizobial adhesion proteins (RapA2, RapB and RapC), and the nodulation protein NodO [94–97]. Glycosyl hydrolases PlyA and PlyB cleave EPS, affecting its processing [97,98]. A *prsD* mutant produces EPS of a higher degree of polymerization than the wild-type strain and elicits a higher number of nodules incapable of fixing nitrogen [94].

### 3.3. Genes Involved in the Synthesis of EPS in Other Rhizobia

In the *S. fredii* NGR234 genome, a 28-kb region comprising *exo* genes organized in four operons has been identified (Figure 2E) [99,100]. These genes are highly homologous to the *exoA, exoB*, *exoY*, *exoL*, *exoM*, *exoN* and *exoP* genes of *S. meliloti*. Large parts of *exo* clusters of *S. fredii* NGR234 and *S. meliloti* species are closely related (especially their *exoX-exoY* regions are almost identical). Additionally, the *exoG* gene, not linked with this *exo* region, has been identified in the *S. fredii* NGR234 genome [101]. The presence of similar *exo* genes in *S. fredii* NGR234 and *S. meliloti* might be explained by the fact that both of these rhizobial species produce EPS of very similar structures (Figure 1C,E). However, some differences in the genetic organization of these *exo* clusters have been observed [102,103]. For example, a non-functional homologue of *S. meliloti exoH*, which is responsible for succinylation of EPS by this bacterium, changed its location from the *exo* cluster in plasmid pNGR234b to *S. fredii* genome [103]. These data explain why, in contrast to *S. meliloti* EPS, EPS of *S. fredii* NGR234 is not succinylated.

A region involved in EPS biosynthesis has also been identified in the genome of *B. japonicum* (Figure 2F) [47,104]. This cluster contains several genes organized into at least four different operons, among them a gene homologous to *S. meliloti exoB*, which encodes a UDP-galactose 4′-epimerase. Protein products of other genes are homologous to UDP-hexose transferases and *S. meliloti* ExoP involved in EPS I chain-length determination. A deletion mutant, which does not contain the DNA fragment encoding the *C*-terminal part of ExoP, the ExoT transferase and the *N*-terminal part of ExoB, synthesizes only the LMW fraction of EPS lacking galactose, nodulates the host plant with a delay and induces symptoms of plant defense reactions [104].

To date, little is known about EPS synthesis in the *Mesorhizobium* genus, encompassing moderately-growing rhizobia. Recently, a cluster involved in the biosynthesis of this polymer has been found in the *M. tianshanense* genome [105]. In this region, the *mtpE* and *mtpABCD* genes were identified, which demonstrate a significant homology to the *R. leguminosarum exo5* and *pssNOPT* genes, respectively. EPS production is completely abolished in both *mtpABCD* and *mtpE* mutants. These mutants also form significantly less biofilm on glass surfaces and are defective in nodulation of their host plant, Asian licorice (*Glycyrrhiza uralensis*) [105].

## 4. Regulation of EPS Biosynthesis

In rhizobia, the process of EPS synthesis is very complex and is regulated at both transcriptional and post-transcriptional levels, with multiple regulatory systems, which up to now have been best studied in two species, *S. meliloti* and *R. leguminosarum*. Adaptation of EPS synthesis to various environmental conditions requires a complex regulatory network involving cross talk between the different regulatory components. Several environmental factors and stress conditions, such as media osmolarity, ammonium and phosphate availability and flavonoids influence EPS biosynthesis [106–108]. Also, other culture conditions, such as the type of carbon source and culture age can modify the amount and composition of EPS produced [109–111].

### 4.1. Regulation of EPS Synthesis in *S. meliloti*

**Figure 3:**
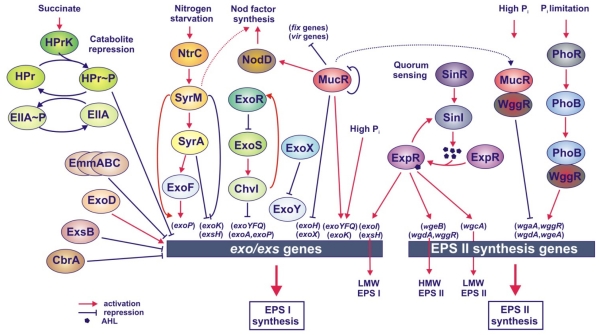
Model of the regulatory network correlating exopolysaccharide production, quorum sensing and catabolite repression in *S. meliloti*.

In *S. meliloti*, several regulatory genes of EPS I and EPS II synthesis have been identified either on the chromosome (*mucR*, *exoR*, *exoS*, *exoD, expR*, *syrM* and *phoB*) or on the megaplasmid pSymB (*exsB*, *exoX* and *wggR*). The majority of the proteins encoded by these genes have been described as repressors (Figure 3). These include the *exoR*, *exoS*, *exoX* and *exsB* genes, which negatively affect EPS I synthesis, and the *mucR*, which negatively regulates EPS II synthesis [57,60,62,107,112]. On the other hand, the SyrM and PhoB proteins are positive regulators of EPS I and EPS II production, respectively [69,113]. Among the identified regulators, MucR seems to be a global regulatory protein playing a key role in both positive regulation of EPS I synthesis and negative regulation of EPS II synthesis, thus coupling these two biosynthetic pathways [113–116].

The production of *S. meliloti* EPS is affected by several nutritional and stress conditions. Limitations of some non-carbon nutrients, like nitrogen and sulfur, very high phosphate concentrations, and hyperosmotic stress stimulate the synthesis of EPS I [61,107]. On the other hand, phosphate starvation stimulates EPS II production [27,107,113], indicating that the concentration of this nutrient is an essential signal affecting which type of EPS will be produced by *S. meliloti*. Also, different osmotic conditions modify EPS biosynthesis in this bacterium. A low osmotic pressure results in the production of mainly LMW EPS I, whereas production of the HMW fraction of this polymer is stimulated by an increased osmotic pressure [117]. Recently, Jofre and Becker [59] have reported that polymerization of EPS I is affected by the ionic strength of the medium rather than osmolarity.

#### 4.1.1. Regulation of EPS I Synthesis

In general, regulation of *S. meliloti* EPS I synthesis is almost negative, with the exception of the MucR and SyrM, which function as positive regulators of this process (Figure 3) [114,116,118]. To date, six regulatory genes have been found to negatively affect EPS I synthesis, including the *exoX* and *exsB* genes linked with the *exo*/*exs* cluster, and the chromosomal *exoR*, *exoS*, *cbrA* and *emmC* genes. Mutants in both *exoR* and *exoS* genes display an increased production of EPS I and a higher expression of several *exo* genes (*exoYFQ*, *exoA* and *exoP*) in relation to the parental strain [61,62,119]. Despite these similarities, they differ in their symbiotic properties; the *exoR* mutant induces non-nitrogen-fixing nodules on alfalfa, whereas the *exoS* mutant elicits the formation of effective nodules on this host plant [62]. The ExoS protein was found to be almost identical to *A. tumefaciens* ChvG, which is a sensor protein of a two-component regulatory system [120]. ExoS is located in the IM in the form of homodimers, and its sensor domain, located in the periplasmic space, is responsible for the recognition of an environmental signal. After the signal has been recognized, ExoS kinase activates a second component of this system, the ChvI response regulator, by its phosphorylation. Next, ChvI affects the transcription of *exo* genes, so that this consequently leads to the modulation of EPS I production (Figure 3) [112]. ExoR is a periplasmic protein, which translocates to the periplasm, where it physically interacts with the ExoS and inhibits ExoS/ChvI two-component signalling [121–123]. *exoR* expression was found to be upregulated in the absence of functional ExoR. This regulation is mediated by the ExoS/ChvI system, which positively affects the transcription of *exoR* [123–125]. Both the ExoR protein and the ExoS/ChvI system, apart from EPS I synthesis, are also involved in the regulation of flagellum biosynthesis genes [112].

Recently, novel *S. meliloti exoS* and *chvI* null mutants have been reported [126,127]. Phenotypic analysis of these two mutants demonstrated that the ExoS/ChvI regulatory system was also required for growth on over 21 different carbon sources. Additionally, the *chvI* mutant exhibited several other effects, such as failure in the growth on complex media, an altered LPS profile, hypermotility, lower tolerance to acidic conditions and synthesis of significantly less poly-3-hydroxybutyrate than the wild type strain. These data indicate that ChvI is a crucial protein engaged in *S. meliloti* regulatory networks involving both the bacterial cell envelope and carbon source utilization [127].

In conclusion, the ExoR, ExoS and ChvI proteins form a regulatory system which appear to be involved in several critical cell processes, such as EPS I production, nutrient utilization, motility and free-living viability [123,126,127].

*exoX* located in the *exo/exs* cluster, is another gene participating in the negative regulation of EPS I synthesis (Figure 3). It has been shown that the ratio of *exoX* copies to the copies of the *exoY* encoding an enzyme, catalyzing the initial step of repeating unit synthesis, is very important for the proper level of EPS I synthesis. ExoX is a small, inner membrane-attached protein showing a significant similarity to the previously identified PsiA of *R. leguminosarum* bv. *phaseoli* and ExoX of *Rhizobium* sp. NGR234 [99,100]. Mutants in *exoX* overproduced EPS I, whereas the presence of multiple copies of this gene inhibited the production of EPS I. Since the expression of *exo* genes was not affected by the gene dosage of *exoX* to *exoY*, it was suggested that ExoX functions as a post-transcriptional inhibitor of the ExoY [56,119]. The fact that both ExoX and ExoY are associated with the IM suggests a direct interaction between these two proteins [100].

*exsB* is a second gene in the *exo/exs* region, which encodes a regulator negatively affecting EPS I synthesis (Figure 3) [57]. Similarly to the phenotype of the *exoX* mutant*,* an *exsB* mutant produced 3-fold more EPS I than the wild type, but in contrast to the *exoX* mutant, additional *exsB* copies resulted in a decrease of EPS I production to 20% of the control. Since this effect was not caused by direct regulation of the transcription of *exo* genes in this mutant background (the only exception was the reduction of *exoK* expression) [60], negative regulation of EPS I production by ExsB must have occurred mainly at the post-transcriptional level [54,57].

Among environmental factors, ammonia has been found to significantly affect EPS I synthesis. The regulation of this process by nitrogen availability is directed by the NtrC and SyrM proteins, which under limitation of this nutrient act as positive regulators. Mutations in genes for these two regulatory proteins decrease EPS I production under nitrogen starvation. The SyrM protein increases the transcription of the *exoF* and *exoP* genes via induction of *syrA* expression, and additionally by direct activation of *exoP* transcription (Figure 3) [118,128]. On the other hand, SyrM inhibits the expression of *exsH* and *exoK* encoding endoglycanases. The *exoK* gene is of special interest among all *exo* genes of the *exo*/*exs* region in that it is a target for the action of at least three regulatory proteins (MucR, SyrM and ExsB), indicating that the level of its expression is very important for the proper degree of polymerization of EPS I.

The SyrM protein is also involved in the regulation of the expression of *nod* genes, which are responsible for the synthesis of another symbiotic signal, the Nod factor [128,129]. This protein belongs to the family of LysR-type transcriptional regulators and displays a similarity to NodD proteins engaged in the activation of *nod* genes [118]. SyrM is involved in the determination of the ratio of LMW to HMW EPS I, and this process is affected by nitrogen and luteolin, the plant flavonoid indispensable for the activation of *nod* genes in the presence of NodD [118,129]. These data indicate that SyrM influences both EPS I and Nod factor synthesis, suggesting that nitrogen starvation might be a factor affecting the concentration of these two signal molecules essential in the early stages of host plant infection.

The precise role of another chromosomal gene, *exoD*, in the regulation of EPS I synthesis is difficult to explain. Nevertheless, the gene seems to positively affect this process because an *exoD* mutant produces diminished amounts of EPS I [63]. Furthermore, this mutant was found to be sensitive to alkaline conditions and elicited effective nodules on host plants exclusively in a slightly acidic growth medium [63].

Among the regulatory proteins of *S. meliloti*, MucR seems to play a key role in the positive regulation of EPS I synthesis. A mutation in *mucR* results in a high level of EPS II production but only a very low level of LMW EPS I synthesis [60,114,115]. The MucR protein contains the C_2_H_2_ type zinc-finger motif and influences the transcription of some *exo* genes (*exoH*, *exoX*, *exoY*) by binding to a palindromic sequence located in promoter regions of the regulated genes, but the observed levels of modulation are not high. In the *mucR* mutant, the transcription of *exoH* and *exoX* was slightly increased, whereas the expression of *exoK* and the *exoYFQ* operon decreased 1.5-fold. This indicates that the positive function of MucR in EPS I synthesis is a result of both stimulation of the expression of EPS I synthesis genes and repression of *exoX* transcription [114,115]. Moreover, MucR negatively regulates its own transcription by binding to a palindromic sequence located in the *mucR* upstream region [60,115].

Recently, Morris and Gonzalez [125] have characterized a novel three-component system, consisting of the EmmA, EmmB and EmmC proteins, which appear to play a crucial role in the adaptability and survivability of *S. meliloti*. EmmA is a protein secreted to the periplasm, whereas EmmB and EmmC are sensor and response proteins, respectively. The *emmABC* genes are associated with EPS I production, motility and stress adaptation. Mutations in the *emm* locus cause motility defects, an increase in succinoglycan synthesis and a decrease in stress tolerance, which, in consequence, leads to the loss of the ability to form an optimal symbiosis with alfalfa [125]. The EmmABC system shows some functional similarity to the ExoR-ExoS-ChvI system, although no cross-regulation of these systems has been evidenced. This suggests that these two systems may act in parallel but separate fashions in the regulation of important cell processes, such as EPS I production and motility, in response to environmental cues.

Moreover, it has recently been demonstrated that the *cbrA* gene, which encodes a stationary phase-induced sensor kinase, is also associated with EPS I synthesis and motility [130]. This is confirmed by the phenotype of the *cbrA* mutant, which overproduces EPS I and shows cell envelope defects, a decrease in flagellar biosynthesis and symbiotic defects.

#### 4.1.2. Regulation of EPS I Synthesis by Succinate-Mediated Catabolite Repression

Recently, succinate-mediated catabolite repression has been characterized in *S. meliloti*, and some proteins of the phosphotransferase system (PTS) engaged in this regulation appeared to affect EPS I production [131,132]. Although this bacterium can utilize a variety of compounds as sources of carbon, succinate is preferred carbon and energy source to other compounds (glucose, fructose, galactose and lactose), playing an especially important role in *S. meliloti* metabolism during both the free-living and symbiotic states. The *hprK*, *EIIA* (*manX*) and *hpr* genes for the PTS system form a cluster in the chromosome located immediately downstream of the *exoS* gene [132]. HPrK of a kinase/phosphatase activity is the main component of the PTS system and it regulates succinate-mediated catabolite repression through phosphorylation/dephosphorylation of its substrate, the protein HPr. A phosphorylated form of HPr activates *EIIA*, that leads to enhancing of catabolite repression in the presence of succinate. A mutation in *hpr* results in several effects, including altered carbon metabolism, production of EPS I, and sensitivity to cobalt limitation [131,132]. An *EIIA* mutant demonstrates slowed growth on diverse carbon sources and enhanced accumulation of HMW EPS I. Despite these strong phenotypes, both *hpr* and *EIIA* mutants exhibit wild-type nodulation and nitrogen fixation on alfalfa [131]. On the other hand, a *hprK* mutant overproduces EPS I and induces nodules that do not fix nitrogen, indicating that this gene, apart from the key role in catabolite repression, is also important for EPS I synthesis and effective symbiosis [132].

#### 4.1.3. Regulation of EPS II Synthesis

Besides nitrogen, phosphate is another environmental factor which plays a significant role in the regulation of EPS synthesis in *S. meliloti*. In contrast to EPS I, whose production is induced in the presence of high phosphate concentrations (above 10 mM), low-phosphate conditions, typically found in soil (0.1 to 10 μM), stimulate EPS II production [19,97,103]. This polysaccharide probably plays a dominant role in the environmental conditions, where this nutrient is scarce. Recently, Rinaudi and Gonzalez [133] have shown that symbiotically active fraction of EPS II is crucial for both root colonization and biofilm formation.

In *S. meliloti*, phosphate regulates a considerable number of genes, including the genes involved in EPS II synthesis, through the PhoB response regulator forming a two-component regulatory system with the sensor kinase PhoR (Figure 3) [67,69,113,134–137]. In general, PhoB functions as a positive regulator which induces the expression of genes belonging to the Pho regulon under P_i_ limitation. In these conditions, a phosphorylated form of PhoB activates the transcription of target genes by binding to the PHO box sequence located in the promoters of phosphate-regulated genes [134,136]. Among genes belonging to the *S. meliloti* Pho regulon, Krol and Becker [135] have found the *wgaA*, *wggR*, *wgdA* and *wgeA* genes involved in EPS II synthesis, whose expression was significantly induced in P_i_-limited cells. In upstream regions of these genes, two PHO box-like sequences have been identified [113,136].

EPS II synthesis is also regulated by two other regulatory proteins, WggR (ExpG) and MucR (Figure 3) [69,138]. WggR, encoded by a gene located in the galactoglucan synthesis gene cluster, positively affects the synthesis of EPS II under P_i_ limitation [67]. This protein, belonging to the Mar family of transcriptional regulators containing a helix-turn-helix motif, binds to conserved palindromic sequences located in promoter regions of the *wgaA*, *wggR*/*wgdA* and *wgeA* genes to activate their expression [69,113]. The deletion of *wggR* reduces EPS II synthesis under P_i_ starvation, whereas additional copies of this gene slightly increase EPS II production [69,113]. The second regulator, MucR, represses galactoglucan synthesis, whereas a mutation in *mucR* leads to the production of the HMW form of this polysaccharide only [60,107,139].

Transcription of the *wga*, *wge* and *wgd* operons is governed by two promoters that are differentially controlled by PhoB, WggR and MucR [69]. Upstream of the distal promoters of these genes, three motifs functioning as binding sites for the MucR, WggR and PhoB proteins have been identified, and the binding site for WggR ovelapped with the PHO box sequence. Under phosphate-sufficient conditions, the transcription of the *wga*, *wgd* and *wge* genes is repressed by MucR, which strongly inhibits the activity of both promoters by binding to the sequence located in the vicinity of the distal transcription start sites, and consequently, only traces of EPS II are produced [69]. The WggR protein also slightly inhibits the activity of the distal promoter under the same conditions. Under P_i_ starvation, WggR and phosphorylated PhoB bind to the promoter regions and activate the transcription of the *wga*, *wgd* and *wge* genes. This phosphate-dependent regulation of EPS II synthesis has been reported to involve the cooperative action of WggR and PhoB. This indicates that WggR plays a special role in the regulation of EPS II synthesis mediated by the PhoB and MucR proteins. The activities of PhoB, WggR and MucR regulators ensure fine-tuning of the expression of galactoglucan synthesis genes, which allows bacteria to adapt to changing environmental conditions [69,113,138]. Moreover, the transcription of *wggR* is activated by PhoB under P_i_ limitation, and both the WggR and PhoB proteins function cooperatively in the transcription activation of this regulatory gene [69].

#### 4.1.4. Role of Quorum Sensing in the Regulation of EPS I and EPS II Synthesis

*S. meliloti* possesses at least three quorum-sensing systems, among them the Sin system, consisting of SinR, a LuxR-type transcriptional regulator, and SinI, an autoinducer synthase responsible for the synthesis of a series of long-chain *N*-acyl homoserine lactons (AHL) (Figure 3) [139,140]. AHL represent one of the classes of pheromone-like signals, called autoinducers, which are released exogenously and whose concentration acts as an indicator of population density for the bacteria. The Sin system regulates the expression of many *S. meliloti* genes, including genes involved in the production of EPS I and EPS II, motility, chemotaxis and other cellular processes, such as nitrogen fixation and metal transport. The regulation of most of these genes is dependent on the presence of another LuxR-type regulator, ExpR [141–144].

*S. meliloti* wild type exhibits a mucoid phenotype due to the presence of EPS II. However, both *sinI* and *expR* mutants remain dry and unable to produce EPS II, which confirms the role of the Sin system and the ExpR protein in the synthesis of this polysaccharide [139,141,142,145]. Expression of *sinI* is dependent on *sinR* and is enhanced by AHL-ExpR (Figure 3). Also, galactoglucan biosynthesis genes (particularly *wgeB*, *wgdA*, *wggR* and *wgcA*) are highly induced in the presence of AHL, and their expression depends not only on the production of this signal but also on the active ExpR protein [139,145]. Expression of genes of the *wge*, *wga* and *wgd* operons is required for the synthesis of HMW EPS II, whereas expression of *wgcA*, encoding glycosyltransferase, is critical for the production of symbiotically active LMW EPS II [145]. Recently, it has been established that ExpR binds to promoter regions of the *sinI*, *wgaA* and *wgeA* genes, and this binding is enhanced in the presence of AHL [142]. Activation of these genes by ExpR is almost completely dependent on the presence of WggR when another regulator, MucR, is present. But in the absence of MucR, WggR is not required for the positive effect of ExpR. This WggR-dependent effect is also seen with PhoB-dependent induction under P_i_ starvation and suggests that WggR functions as a general mediator of the expression of EPS II synthesis genes for at least two regulators (PhoB and MucR) under different conditions [69]. The close proximity of the binding sites for ExpR, WggR, PhoB and MucR suggests a complex regulation of EPS II synthesis genes involving protein-protein interaction and, possibly, some competition for the target sites, which allows changing the level of production of this polymer in response to different environmental factors [69,142]. MucR appeared to repress EPS II synthesis at an extremely low population density until the ExpR/Sin system abolishes this effect at levels of quorum sufficient for biofilm formation and invasion of the host plant [144].

Moreover, ExpR and the Sin quorum-sensing system regulate some *exo/exs* genes involved in EPS I production (Figure 3) [142,143,146]. ExpR stimulates the expression of *exoI* and *exsH* encoding a succinoglycan glucanase which is responsible for the generation of the LMW form of this polysaccharide [143]. In the promoter regions of these genes, binding sites for ExpR have been found [142]. These data confirm that the ExpR/Sin system is engaged in the regulation of not only EPS II, but also EPS I biosynthesis.

The regulation of EPS synthesis by *sinRI* is an important finding, which connects quorum sensing with the symbiotic process. A *sinI* mutant unable to produce AHL is deficient in invasion of alfalfa plants, indicating that the Sin system plays an essential role in the establishment of an effective symbiosis [143].

Recently, Mueller and Gonzalez [144] have reported that MucR plays a more global regulatory role, being engaged, together with the ExpR/Sin system, in the complex regulation of symbiotic functions. In planctonic *S. meliloti*, MucR properly coordinates a diverse set of bacterial behaviors by repressing a variety of genes, whose expression is activated only during symbiosis, including those involved in nitrogen fixation and respiration (*fix* genes) (Figure 3). In addition, MucR has been found to enhance the bacterial ability to induce nodule formation on the host plant by increasing Nod factor synthesis via increased expression of *nodD* [144].

#### 4.1.5. Roles of the MucR, ExoR/ExoS/ChvI and ExpR/Sin Systems in the Regulation of Motility Genes

**Figure 4:**
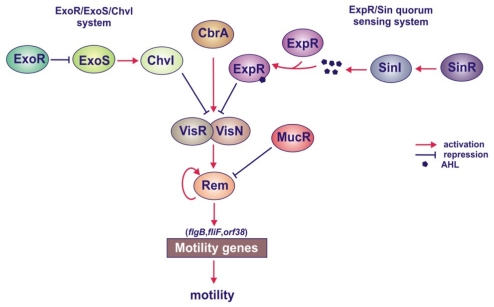
The regulation of *S. meliloti* motility genes by MucR, CbrA and two ExoR/ExoS/ChvI and ExpR/SinR/SinI regulatory systems.

Bacterial motility is one of the critical factors for the establishment of symbiosis under natural soil conditions. The regulatory proteins MucR, ExoR and ExpR, which play an essential role in EPS synthesis, appear to be also involved in motility, indicating that the regulation of these two processes is coupled in *S. meliloti* (Figure 4) [112,116,141,144,147–149]. The 56-kb chromosomal region, named the flagellar region, encompasses a majority of genes associated with motility. These genes, categorized into three main classes, are expressed in a hierarchical manner [148,149]. At the top of the hierarchy are *visN* and *visR* genes, which encode subunits of the LuxR-type transcriptional activator of motility and chemotaxis genes [148,149]. The VisN/VisR regulator activates the transcription of *rem*, which codes for a protein postively regulating the expression of the motility and chemotaxis genes (Figure 4). A disruption of *visN*, *visR* or *rem* leads to the loss of flagella and, in consequence, the loss of swimming and swarming motility. Rem positively regulates the expression of its own gene and the expression of motility genes by direct binding to the promoters of *flgB*, *fliF* and *orf38* [141,147]. Tambalo and associates have described recently that VisN/R-Rem cascade also participates in the regulation of a majority of flagellar, motility and chemotaxis genes (*che1*, *motA*, *flaABCD*, *motB* and *mcpD*) in *R. leguminosarum* [150,151]. All of these genes except *mcpD* are located within the main motility and chemotaxis gene cluster of *R. leguminosarum*. However, other chemotaxis and motility genes (*che2* operon, *flaH*, *flaG*, *flaE* and *mcpC*) found outside of this cluster are not regulated by VisN/R and Rem activators, suggesting a role of additional regulatory system in this process.

Recently, other regulators of motility have been found, which are encoded by genes located outside this region, among them CbrA, initially described as a regulator of symbiosis [130], MucR, and the two systems, ExpR/Sin and ExoR/ExoS/ChvI (Figure 4) [112,116,130,144,147]. A lack of functional CbrA results in a decreased expression of several motility genes, among them *visN*, *visR* and *rem* [130]. Hoang and associates [147] demonstrated that the ExpR/Sin system controls motility gene expression via the VisN/VisR and Rem regulators in a population-density-dependent manner. This system suppresses motility by repression of *visN* and *visR* expression when the bacteria reach the late logarithmic growth phase [139]. Also, the second regulatory system, ExoR/ExoS/ChvI, which is involved in the regulation of EPS I synthesis, suppresses motility gene expression through VisN/VisR and Rem [147]. In both *exoR* and *exoS* mutants, the expression of all genes for flagellum biosynthesis was downregulated, and for this reason, the cells carrying these mutations lost the ability to swim and swarm [112]. Recently, MucR has also been found to participate in the regulatory network controlling motility in *S. meliloti* [116]. This protein, apart from activating EPS I and inhibiting EPS II synthesis, downregulates the expression of *rem* by direct binding to its upstream region.

In conclusion, all three regulatory pathways mediated by the MucR protein and the ExoR/ExoS/ChvI and ExpR/Sin regulatory systems suppress motility through repression of the expression of the regulatory *visN*, *visR* and *rem* genes [112,116,144,147].

### 4.2. Regulation of EPS Synthesis in *R. leguminosarum*

**Figure 5:**
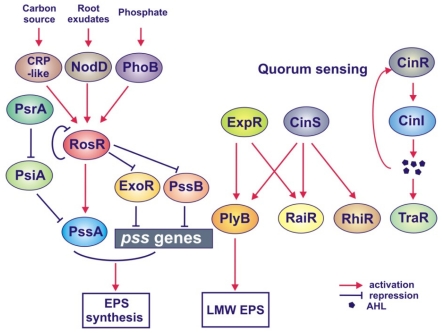
Model of regulation of EPS synthesis in *R. leguminosarum*.

In contrast to *S. meliloti*, the knowledge about regulation of EPS biosynthesis in *R. leguminosarum* is much more fragmentary. Up to now, only a few regulatory genes involved in this process have been described. These include two genes, *psiA* and *psrA*, located on the symbiotic megaplasmid (pSym), and *exoR*, *pssB*, *rosR* and *expR* genes located on the chromosome of *R. leguminosarum* (Figure 5) [82,83,152–156].

*psiA* (a polysaccharide inhibition gene) and *psrA* (a polysaccharide restoration gene), the first to be identified among those genes, have been found on pSym of *R. leguminosarum* bv. *phaseoli*, near the *nod-nif* region involved in nodulation and nitrogen fixation [152]. Although a mutation in *psiA* does not affect EPS production, additional copies of this gene prevent EPS synthesis and abolish nodulation of *Phaselous* plants [81–83,152]. The inhibitory effect of extra *psiA* copies is overcome in the presence of multiple copies of *psrA* or *pssA*, encoding the glucosyl-IP-transferase engaged in the first step of EPS synthesis [81,82]. This indicates that for proper EPS production, a balanced number of *psiA*, *pssA* and *psrA* copies is required. *psiA* encodes a small, inner membrane-attached protein, which shows a similarity to the *S. meliloti* ExoX regulator. Also PssA, containing a hydrophobic *N*-terminal end and a transmembrane helix domain is located in the IM [81,157]. The same subcellular localization of both PsiA and PssA proteins suggests that PsiA most probably functions as a post-translational inhibitor of PssA, which binds to and inhibits the activity of this enzyme.

The second regulator, PsrA, which belongs to a family of transcriptional regulators containing a helix-turn-helix motif represses the transcription of *psiA* (Figure 5) [83,152]. A *psrA* mutant produces a decreased amount of EPS in comparison to the wild type strain, suggesting a positive role of this gene in EPS synthesis. Both a strain with multiple copies of *psrA* and a *psiA* mutant demonstrate similar symbiotic phenotypes—they induce the formation of non-nitrogen-fixing nodules on the host plant. These data suggest that *psiA* most probably inhibits EPS production inside nodules via the repression of *psrA.* The *psiA* and *psrA* genes have been identified exclusively on pSym plasmids of *R. leguminosarum* bv. *phaseoli* strains, indicating that this regulatory mechanism is specific only to this biovar [78,152].

EPS production in *R. leguminosarum* is also negatively regulated by *exoR*, which shows a significant similarity to the *exoR* of *S. meliloti* (Figure 5). An *R. leguminosarum* bv. *viciae exoR* mutant produces 3-fold more EPS than the wild-type strain similarly to the *S. meliloti exoR* mutant [153]; but in contrast to this latter mutant, it induces both effective and non-infective nodules on pea plants.

Also *pssB*, located upstream of the *pssA* gene, seems to be engaged in negative regulation of EPS synthesis; however, its precise role in this process is difficult to establish. A *pssB* mutant produces higher amounts of EPS than the wild type and induces non-nitrogen-fixing nodules on clover and vetch [154,158,159], whereas additional copies of this gene result in increased EPS production [160]. *pssB* encodes a protein belonging to a family of inositol monophosphate phosphatases (IMP-ases), encompassing enzymes from both prokaryotic and eukaryotic organisms [159,160]. In mammalian cells, IMPases are responsible for the conversion of inositol monophosphate to inositol, which is needed for the regeneration of phospholipids containing inositol. But the role of IMPase in the metabolism of rhizobia is unclear. This enzyme may generate the pool of inositol, which is a compound commonly found inside bacteroids of *R. leguminosarum* bv. *viciae* and also abundantly occurring in pea nodules. On the other hand, inositol catabolism seems to be important for survival and competition of rhizobial strains, being crucial for successful invasion of host plants [160].

#### 4.2.1. Role of RosR in EPS Synthesis and Symbiosis

Recently, *rosR*, which seems to play a key role in the regulation of EPS synthesis among all the so far described regulatory genes, has been identified on the chromosome of *R. leguminosarum* bv. *trifolii* (Figure 5) [155]. This gene is very conserved and is present in the genomes of all strains belonging to three *R. leguminosarum* biovars and closely related species, *R. etli* and *R. gallicum*, suggesting its important regulatory role in other rhizobial species as well [78,161]. *R. leguminosarum rosR* also shares a significant identity with *ros* of *A. tumefaciens* [162], *rosAR* of *A. radiobacter* [163] and *mucR* of *S. meliloti* [60]. All these genes encode transcriptional regulators belonging to the family of Ros/MucR proteins, which possess C_2_H_2_ type zinc-finger motifs and are involved in the regulation of EPS synthesis. A genome-wide genetic screening has shown that *R. etli* RosR affects the expression of many functionally diverse genes, among them those responsible for the synthesis and modification of surface polysaccharides (*exoB*, *prsD*, *pssK* and *plyA*) [164]. A mutation in *R. leguminosarum* bv. *trifolii rosR* results in a substantial decrease of EPS production and ineffective symbiosis with clover, whereas additional copies of this gene lead to a nearly twofold increase of EPS synthesis [79,155], indicating that *rosR* functions as a positive regulator of this process. Moreover, a *rosR* mutant exhibits several other effects, including a decreased ratio of LMW to HMW fractions of EPS, quantitative alterations in the polysaccharide constituent of LPS, changes in membrane and secreted protein profiles, higher sensitivity to surface-active detergents and some osmolytes, and decreased motility and nodulation competitiveness [165]. But the most striking effect of *rosR* mutation is the considerably decreased attachment and colonization of root hairs, indicating that the mutation affects the first steps of the invasion process. On the other hand, multiple *rosR* copies significantly enhance competitiveness and clover nodulation in *R. leguminosarum* bv. *trifolii* strains, confirming the essential role of this gene in symbiosis [79].

The data obtained by our research group indicate that *rosR* expression is very complex and is modulated by different environmental factors (Figure 5) [108,155,166]. This gene demonstrates a very high level of expression, which is due to the action of its strong distal promoter as well as two additional regulatory elements, an upstream promoter element and TGN extended −10 element [166]. In addition, several other sequence motifs were identified in the *rosR* upstream region, among them a LysR motif recognized by proteins from the LysR family, motifs resembling *E. coli* cAMP-CRP binding site, PHO boxes as binding sites for the PhoB regulator, and the RosR box [155,166]. The complexity of *rosR* regulation is additionally enhanced by six inverted repeats of different lengths found in this region, which are most probably engaged in post-transcriptional regulation, affecting the stability of *rosR* transcripts [108]. We confirmed that RosR recognizes and binds to the 22-bp-long palindromic sequence, called the RosR box, and decreases the transcription of its own gene [155]. In the presence of glucose, *rosR* transcription is also significantly decreased, indicating that the expression of this gene is regulated by catabolic repression [166]. Moreover, *rosR* transcription increases in the presence of clover root exudates and the functional activator NodD [108]. Also phosphate affects *rosR* expression, and PHO boxes located in the *rosR* upstream region are most probably target sites for PhoB. These data show that four regulatory proteins (RosR, CRP-like protein, NodD and PhoB) are involved in the regulation of *rosR* expression in response to different environmental factors (Figure 5). Also, EPS production in *R. leguminosarum* is affected by phosphate and ammonia, plant flavonoids and the type of carbon source [108,166,167]. This indicates that the increase of EPS synthesis in the presence of flavonoids and glycerol, and under phosphate limitation, is mediated by *rosR*, whose transcription also increases significantly in these conditions. The positive role of RosR in EPS production seems to be a coupled effect of stimulation of *pssA* expression [155] and repression of the expression of the *exoR* and *pssB* genes, engaged in the negative regulation of this process (Figure 5) [168]. In addition, root exudates, phosphate and ammonia starvation affect the transcription of some *pss* genes (*pssA*, *pssB*, *pssO* and *pssP*) [79,169].

In general, a positive effect of flavonoids and *nod* genes on EPS production has been observed in *R. leguminosarum* and *S. meliloti*, suggesting connected regulation of the synthesis of both symbiotic signals, EPS and Nod factors. But, influence of flavonoids on EPS production seems to be different in different rhizobia, because the presence of genistein—an effective inducer of *nod* genes in *S. fredii* HH103, results in a non-mucoid phenotype [170,171]. On the other hand, *nolR* encoding a regulator of *nod* genes has a positive effect on EPS synthesis in this strain [170,171].

#### 4.2.2. Role of Quorum Sensing in the Regulation of EPS Synthesis

EPS production in *R. leguminosarum* also seems to be influenced by quorum sensing (Figure 5) [140]. In *R. leguminosarum* bv. *viciae*, four different AHL-based quorum sensing systems, *cin*, *rai*, *rhi* and *tra*, have been discovered, which are involved in several processes, including plasmid transfer and nodulation [172]. The *cinR*, *cinI* and *cinS* genes of the *cin* system are at the top of this hierarchical regulatory network and induce the other (*rai*, *rhi* and *tra*) systems. CinI is responsible for the production of *N*-(3-hydroxy-7-*cis-*tetradecenoyl)-l-homoserine lactone, and CinR induces *cinI* in response to this AHL [140]. The third element of the *cin* system, CinS, is a small protein having an anti-repressor function, which induces the regulatory genes *rhiR* and *raiR* of the *rhi* and *rai* systems, and also *plyB* encoding an extracellular glycanase (Figure 5) [156,173]. This protein acts by attenuating the repressive activity of another regulator, PraR. A cloned *cinS* causes an apparent loss of colony mucoidy with colony aging, and this unusual phenotype is a result of the action of increased amounts of PlyB, which degrades EPS. Another protein that is required for the induction of both *raiR* and *plyB* is ExpR, which functions independently of CinI-made AHL [156,173]. *R. leguminosarum expR* is homologous to the *expR* of *S. meliloti* and the two proteins encoded by these genes are LuxR-type regulators. Nevertheless, several functional differences between them have been found. In contrast to the *expR* mutant of *S. meliloti*, the colony morphology of the *R. leguminosarum expR* mutant is very similar to the wild type. This indicates that *expR* regulates a lower number of genes in *R. leguminosarum* than its homolog from *S. meliloti*, and among them only one gene, *plyB*, which is associated with EPS production [156,173].

### 4.3. Role of EPS Synthesis Genes in Biofilm Formation in Rhizobia

Similarly to other bacterial EPS, rhizobial EPS play a significant role in biofilm formation, being the major components of its matrix, which provides a physical barrier against diffusion of toxic compounds and protection against environmental stresses [17]. A mutation in *R. leguminosarum pssA*, a key gene for EPS synthesis, completely abolishes the production of this polysaccharide and biofilm formation [174]. Also, the size of EPS is very important for normal biofilm development, as borne out by the fact that *prsD*, *prsE*, *plyB* and *plyBplyA* mutants, which produce EPS of significantly longer chains than the wild type, are impaired in biofilm formation [174]. Both mutants in the *prsD* and *prsE* genes encoding components of the PrsD-PrsE type I secretion system are only able to form biofilm of an immature structure. In addition, mutants in the *plyA* and *plyB* genes, which encode glycanases cleaving EPS, show delayed formation of a biofilm with an atypical structure. Also, RapC, RapA1 and RapA2 agglutinins secreted via the PrsD-PrsE system are responsible for the adhesion and aggregation of rhizobia [4,174]. Moreover, a mutation in *rosR* results in the formation of lower amounts of biofilm, which differs significantly in depth, architecture and viability of cells from that of the wild type, confirming the important role of this gene in biofilm formation [108,165]. Similarly in *S. meliloti*, EPS I is required for biofilm development, since an *exoY* mutant non-producing this polysaccharide forms an immature biofilm [175]. Rinaudi and Gonzalez have reported [133] that the ExpR/Sin quorum-sensing system controls biofilm formation in this bacterium through the production of EPS II. The symbiotically active LMW fraction of this polymer appeared to be a crucial factor for the development of biofilm and root colonization [133]. Also, the *cin* quorum system is engaged in biofilm formation by *R. leguminosarum*, because a *cinS* mutant forms higher amounts of biofilm than the wild type [173].

Moreover, several nutrients and stress conditions regulate biofilm formation by *S. meliloti* and *R. leguminosarum.* In *S. meliloti*, the concentrations of sucrose, calcium and phosphate positively correlate with biofilm formation, whereas extreme temperature and pH values have a negative effect on this process [176]. Also, nutrients such as phosphate, ammonium, type of carbon source and plant flavonoids affect biofilm formation by *R. leguminosarum* [108]. This finding could be explained, to some extent, by the increased production of EPS in these growth conditions. Besides EPS, other rhizobial polysaccharides and components are also involved in biofilm maturation, including Nod factors and flagellum, pointing to the complexity of this process [17]. Flagella-less mutants of *S. meliloti* exhibit reduced biofilming capabilities [175]. Also, common *nodD1ABC* genes engaged in the synthesis of core Nod factor are required for the establishment of a mature biofilm by *S. meliloti* [177]. Williams and co-workers described that, apart from EPS, other polysaccharides are important for attachment and biofilm formation by *R. leguminosarum* [178]. It has been indicated that cellulose (*celA*) and glucomannan (*gmsA*) mutants of *R. leguminosarum* do not form biofilms on root hairs, although they develop normal biofilms *in vitro*, confirming a significant role of these polymers in bacterium-plant interactions.

## 5. Conclusions

Extracellular polysaccharides secreted in large amounts by rhizobia are species-specific polymers which, for many years now, have been a subject of great interest because of their important role in successful nodulation of leguminous plants. For this reason, many data concerning the synthesis of rhizobial EPS and regulation of this process by environmental factors have been obtained, especially for the species *S. meliloti*. It has been demonstrated that EPS synthesis in this bacterium undergoes very complex regulation, and its link with other cellular processes (motility, quorum sensing, catabolite repression) essential for survival in soil, colonization and infection of host plants has been confirmed. In contrast to *S. meliloti*, the knowledge about the function of the particular enzymes and regulatory proteins required for EPS synthesis in other rhizobial species is still fragmentary. The recently obtained genome sequences of several rhizobial species will advance functional analysis of the genetic regions involved in the synthesis of surface polysaccharides. Another unexplored area is recognition of LMW EPS, the biologically active form indispensable for the establishment of an effective symbiosis. In spite of intensive studies, the mechanism of the action of this signal molecule and its interaction with plant receptors has not been discovered so far.

## Abbreviations

Glc: glucose
GlcA: glucuronic acid
Gal: galactose
Man: mannose
Rha: rhamnose
OAc: acetyl
Pyr: ketal pyruvate
Suc: succinyl
OHb: hydroxybutanoyl
Me: methyl groups
